# How scientists perceive the evolutionary origin of human traits: Results of a survey study

**DOI:** 10.1002/ece3.3887

**Published:** 2018-02-26

**Authors:** Hanna Tuomisto, Matleena Tuomisto, Jouni T. Tuomisto

**Affiliations:** ^1^ Department of Biology University of Turku Turku Finland; ^2^ National Institute for Health and Welfare Kuopio Finland

**Keywords:** alternative hypotheses, aquatic ape hypothesis, bipedalism, encephalization, human evolution, nakedness

## Abstract

Various hypotheses have been proposed for why the traits distinguishing humans from other primates originally evolved, and any given trait may have been explained both as an adaptation to different environments and as a result of demands from social organization or sexual selection. To find out how popular the different explanations are among scientists, we carried out an online survey among authors of recent scientific papers in journals covering relevant fields of science (paleoanthropology, paleontology, ecology, evolution, human biology). Some of the hypotheses were clearly more popular among the 1,266 respondents than others, but none was universally accepted or rejected. Even the most popular of the hypotheses were assessed “very likely” by <50% of the respondents, but many traits had 1–3 hypotheses that were found at least moderately likely by >70% of the respondents. An ordination of the hypotheses identified two strong gradients. Along one gradient, the hypotheses were sorted by their popularity, measured by the average credibility score given by the respondents. The second gradient separated all hypotheses postulating adaptation to swimming or diving into their own group. The average credibility scores given for different subgroups of the hypotheses were not related to respondent's age or number of publications authored. However, (paleo)anthropologists were more critical of all hypotheses, and much more critical of the water‐related ones, than were respondents representing other fields of expertise. Although most respondents did not find the water‐related hypotheses likely, only a small minority found them unscientific. The most popular hypotheses were based on inherent drivers; that is, they assumed the evolution of a trait to have been triggered by the prior emergence of another human‐specific behavioral or morphological trait, but opinions differed as to which of the traits came first.

## 1 INTRODUCTION

Human evolution is a topic that interests not just researchers specialized in paleoanthropology, but also other scientists and the general public. A number of conflicting hypotheses have been put forward to explain why humans have become strikingly different from other primates. Most scientists in relevant fields (such as paleoanthropology, paleontology, ecology, evolution and human biology) have never published their views on the drivers of human evolution in general, nor on which of the proposed hypotheses on the origin of specific human traits they find most substantiated. No recent summary of the mainstream view among paleoanthropologists has been published either, so there is uncertainty as to whether scientists agree on the driving forces behind human evolution or not. The idea of carrying out a survey to find out emerged when one of us was teaching a university course on human evolution, happened to check what Wikipedia had to say on the subject, and noticed that some Talk pages (especially the one behind the article “Aquatic ape hypothesis”) contained definite but unreferenced claims about what the opinions of “all scientists” or “all paleoanthropologists” are.

**Figure 1:**
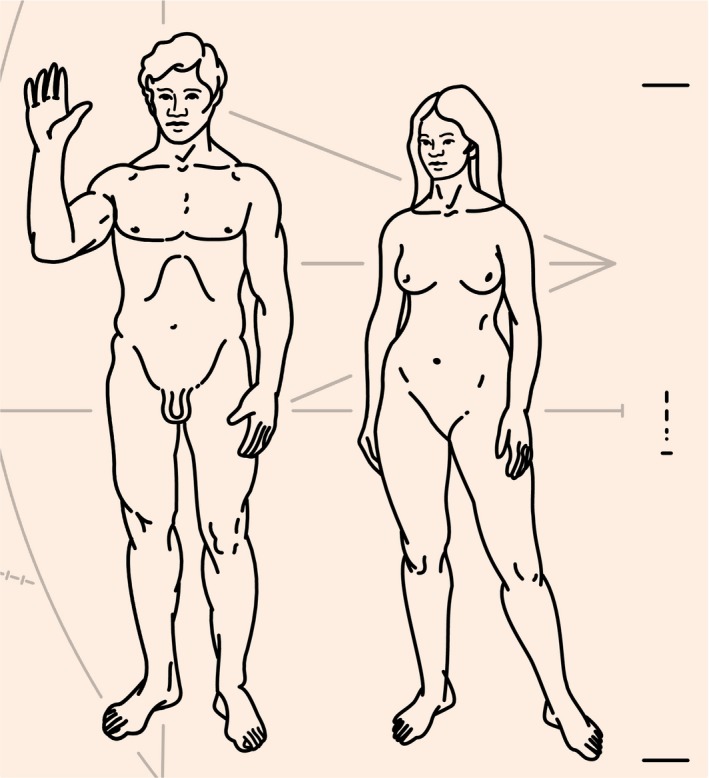
Male and female human figures from the plaque of the Pioneer 10 and 11 spacecrafts. The pictorial message was intended to describe the origin of the probe for potential extraterrestrial life. It shows several typically human traits, such as bipedalism, nakedness, arched nose, large head, and opposable thumbs. Source: NASA; vectors by Mysid (Public domain), via Wikimedia Commons

Humans differ from all the other 400 primate species in many respects, some of the most striking ones being that they walk fully upright on their hind legs, have unusually big brains, and have an effectively naked rather than fur‐covered skin (Figure 1). Other features that among primates are uniquely human include descended larynx, articulated speech and the capacity to accumulate fat in a thick subcutaneous layer.

A number of conflicting hypotheses have been proposed to explain why these and other traits originally evolved in the lineage leading to humans but in none of the lineages leading to other extant primates. One line of argumentation is based on the widely accepted idea that animal species adapt to their environment by natural selection: Traits that give the animal a higher probability of survival and reproduction become more common over time and traits related to lower survival and reproduction rates become less common. Adaptive traits are often morphological (like long legs that increase running speed and facilitate escaping from predators, or thick fur that protects from heat loss in cold weather), but they can also be behavioral (like building a nest or being nocturnal). The corollary of viewing traits of a species as adaptations to its environment is that traits are expected to change if the environment changes, because then also the adaptive pressures change. In particular, if sister species have very different traits in spite of close genetic relatedness, the adaptationist scenario suggests that the lineages experienced different environments during their evolutionary past.

It has indeed been proposed that the ancestors of humans came to live in a different kind of environment than the ancestors of chimpanzees and gorillas, and adapted by evolving a suite of novel traits. One of the early proposals along these lines, suggested already by Lamarck and Darwin, was that human ancestors descended from the trees and moved to the open savanna (Bender, Tobias, & Bender, 2012; Dart, 1925; Domínguez‐Rodrigo, 2014; Leakey & Lewin, 1977). Because terrestrial life in the dry savanna is very different from arboreal life in wet forests, this change in habitat would have shifted the prevailing selection pressures: Traits that were adaptive in the old environment could become maladaptive in the new one, and novel morphological traits could be favored if they gave a higher probability of survival and reproduction. The ancestors of the great apes stayed in the forest and, therefore, remained more similar to other primates.

The savanna scenario has lost some of its appeal since paleoenvironmental reconstructions started to show that the environmental setting has been more complex than was originally thought. Accordingly, more recent accounts describe the environment of early human ancestors as a mosaic of woodlands, savanna, and water bodies with considerable temporal fluctuations between climatically arid and wet periods (Bender et al., 2012; Domínguez‐Rodrigo, 2014; Kingston, 2007; Kovarovic & Andrews, 2007; Maslin & Christensen, 2007). Environmental variability itself has also been proposed to have selected for versatility of adaptations (Potts, 1998a,b).

There have been different views on which aspects of terrestrial life would have required the morphological changes that the human lineage has experienced, so a large number of different explanations have been put forward for each trait. For example, the origin of the bipedal gait has been attributed to (among other things) gaining better visibility over the savanna grass (Ravey, 1978), reaching for food on low branches (Hunt, 1994, 1996), collecting small food items from the ground (Jolly, 1970; Kingdon, 2003), exposing a smaller part of the body to the scorching sun (Wheeler, 1984, 1991), allowing more energy‐efficient long‐distance travel (Carrier et al., 1984; Pontzer, Raichlen, & Sockol, 2009; Rodman & McHenry, 1980), and freeing the hands to carry food, tools, weapons, or babies (Bartholomew & Birdsell, 1953; Hewes, 1961; Lovejoy, 1981; Sutou, 2012; Washburn, 1960). It has also been proposed that bipedalism originated already in the trees for hand‐supported walking on small branches too weak for brachiation (Crompton, Sellers, & Thorpe, 2010; Thorpe, Holder, & Crompton, 2007).

Another adaptationist proposal is that the human ancestors moved from the trees to the waterside, and started to adapt to a partly aquatic way of life (Hardy, 1960; Morgan, 1982; Verhaegen, Puech, & Munro, 2002). This would have exposed them to similar selection pressures than semi‐aquatic mammals, rather than to selection pressures typically experienced by other primates. Under this scenario, bipedal gait would have emerged because it allowed wading to deeper water and made the body more streamlined when swimming and diving for food (Kuliukas, 2002; Morgan, 1990; Niemitz, 2010; Verhaegen et al., 2002).

Not all traits need to have originated to enhance survival, however, and critical voices have been raised against interpreting all uniquely human traits as adaptations driven by natural selection (Gee, 2013). Sexual selection is known to have produced spectacular new traits in various animals, typically ornaments whose sole purpose is to attract the attention of the opposite sex. These confer no survival advantage or may even be harmful to the bearer. At least human bipedalism, nakedness, and subcutaneous fat layer have been explained by this mechanism (Barber, 1995; Giles, 2011; Tanner, 1981). Especially in small populations, traits may even emerge due to chance fixation of random variation (Sutou, 2012).

For someone interested in the “why” of human evolution, it is currently hard to find a comprehensive account of the scientific state of the art. Journal articles typically address only one or a few hypotheses in isolation of the others and often their focus is more on “how” than on “why” a given trait originally emerged (e.g., Crompton et al., 2010; Cunnane & Crawford, 2014; Isler & Van Schaik, 2014; Stout & Chaminade, 2012; Watson, Payne, Chamberlain, Jones, & Sellers, 2008; Wells, 2006). Only proponents of the aquatic/waterside hypotheses (collectively known as the aquatic ape hypothesis or AAH) seem to maintain that it is possible to explain most of the uniquely human traits as adaptive responses to a specific external factor (e.g., Morgan, 1997; Vaneechoutte, Kuliukas, & Verhaegen, 2011), but these views have found little resonance in paleoanthropological journals (Bender et al., 2012). Indeed, AAH has been fiercely opposed and criticized for being an umbrella hypothesis that attempts to explain everything, for being unparsimonious, for lacking evidence and even for being pseudoscience (Hawks, 2005; Langdon, 1997; Moore, 2012).

Here, we aim to find out what scientists really think about why some of the most striking human traits have emerged. We do so by analyzing the results of an online survey where scientists were directly asked for their views on the issue.

## 2 MATERIALS AND METHODS

### 2.1 Survey

A survey was performed using an online form in early 2013. Invitation to participate in the survey was sent by email to the authors of articles and review papers that had been published in a scientific journal of a relevant field during the three previous years (2010–2012). A 3‐year period was thought to be long enough for most researchers to have published at least one scientific paper, but short enough for most of the email addresses given in those papers not to have become obsolete. The focus was on journals of paleontology, zoology, ecology, evolutionary biology, and human biology. Only journals with an ISI impact factor equal to or larger than 1.0 were considered. The exact criteria used to select the journals, as well as a full list of journal names, can be found in Appendix S1.

Almost 58,000 unique email addresses were found in the information available online for the papers published in the selected journals during the selected time period. The full address list exceeded the capacity of the online survey system (Webropol), so the addresses were sorted in alphabetical order, and an invitation to participate in the survey was sent to the first 29,000 addresses. The remaining addresses were used for a different survey, whose results will be reported elsewhere. The first page of the online survey informed participants about the purpose of the survey. The survey was performed anonymously, and all who responded did so voluntarily. After a few reminders had been sent, a total of 1,266 persons had submitted their responses to the survey.

Although the initial sample was large and can be considered representative of the scientific community in relevant fields, the proportion of invitees who answered the survey was very small (4.4%). The sample is no doubt biased toward people who have a larger than average interest in human evolution. Therefore, the obtained answers do not reflect the opinions of the entire scientific community. Nevertheless, they can indicate whether any of the hypotheses proposed to explain the evolutionary origin of a specific human trait is universally accepted or rejected. Even if this were not the case, the survey gives indication of which hypotheses are most or least popular, although conclusions in this respect remain tentative.

The survey first asked background information of the respondent, such as gender, age, the highest academic degree obtained, number of scientific publications authored (both overall and on human evolution), degree of knowledge about human evolution, and whether the respondent has taught courses on human evolution. The second part listed fifteen human traits (such as bipedalism) and asked the respondents to rate the credibility of 51 alternative hypotheses that have been proposed to explain their evolutionary origin (such as freeing the hands for tool use or seeing over tall grass). The credibility scoring was done using a five‐point scale: very unlikely, moderately unlikely, no opinion, moderately likely, and very likely. The number of alternative hypotheses considered was ten for both bipedalism and brain size, eight for hairlessness, seven for speech, four for subcutaneous fat, and three for descended larynx. In addition, there were nine traits for which only one explanation has been proposed in the literature, and this was related to the aquatic ape hypothesis. The third part asked about the respondents’ views on criticism against AAH. All questions and a summary of the answers are presented in Appendix S2.

### 2.2 Data analyses

The respondents were asked for their professional field of expertise by offering 15 alternatives. For statistical analyses, these were simplified to four categories to ensure sufficient sample size in each. The group “(paleo)anthropologist” was formed by lumping the originally separate fields “paleoanthropology” and “anthropology or archaeology.” The group “biologist” was formed by lumping all the original subfields of biology (animal physiology, anatomy, or morphology; ecology; evolution; genetics or molecular biology; other) and the group “human biologist” by lumping all subfields of human biology (cardiovascular or respiratory system, musculoskeletal system, nervous system, nutrition, other aspects of human biology). The fourth group was “other,” which contained the remaining fields (geology, paleontology, other).

Overall relationships among the hypotheses were visualized by principal coordinates analysis (PCoA), where the objects were the hypotheses and the descriptors were individual respondents, with the variable of interest being the credibility score each respondent had given to each hypothesis. A Euclidean distance matrix was calculated, such that the distance between two hypotheses reflects how differently the respondents scored their credibilities. Every respondent who gave one of the hypotheses a higher score than the other increased the final distance between the hypotheses, with the overall distance between the hypotheses equaling zero if every respondent had scored both hypotheses similarly (irrespective of whether the score itself was high or low). PCoA visualizes these pairwise distances, so the closer together two hypotheses get plotted in the ordination diagram, the more similar their explanatory value is in the opinion of an average individual respondent.

The respondents themselves were plotted in the PCoA ordination space on the basis of the scores they had given to the hypotheses. Therefore, the relative positions of the respondents reflect their opinions on the hypotheses: Respondents get plotted toward the same part of the ordination space as the hypotheses they gave highest credibility scores, and far away from the hypotheses they gave lowest scores.

Relationships between the respondents’ opinions and their backgrounds were first assessed visually with the help of the ordination diagram. We then used analysis of variance to test whether there were differences in the average opinions of respondents of different backgrounds. If so, a post hoc Tukey's honest significance test was carried out to assess which aspects of the respondents’ background were associated with differences in opinion. A more detailed breakdown of the respondents’ opinions was obtained by visually comparing the distributions of the credibility scores given to the different hypotheses. This was done both to obtain an idea of which hypotheses are most popular overall, and to see if there were differences among respondents representing different scientific fields and/or having different levels of scientific experience.

R statistical software version 3.3.2 (https://cran.r-project.org/) was used both to run the analyses and to produce the graphs. The vegan package (Oksanen et al., 2015) was used for principal coordinates analysis. The survey data and all R code used to manipulate and analyze the data are available at Opasnet web‐workspace http://en.opasnet.org/w/Evolutionary_origin_of_human_traits. The survey data are also available from the Dryad Digital Repository https://doi.org/10.5061/dryad.s9r98.

## 3 RESULTS

**Figure 2:**
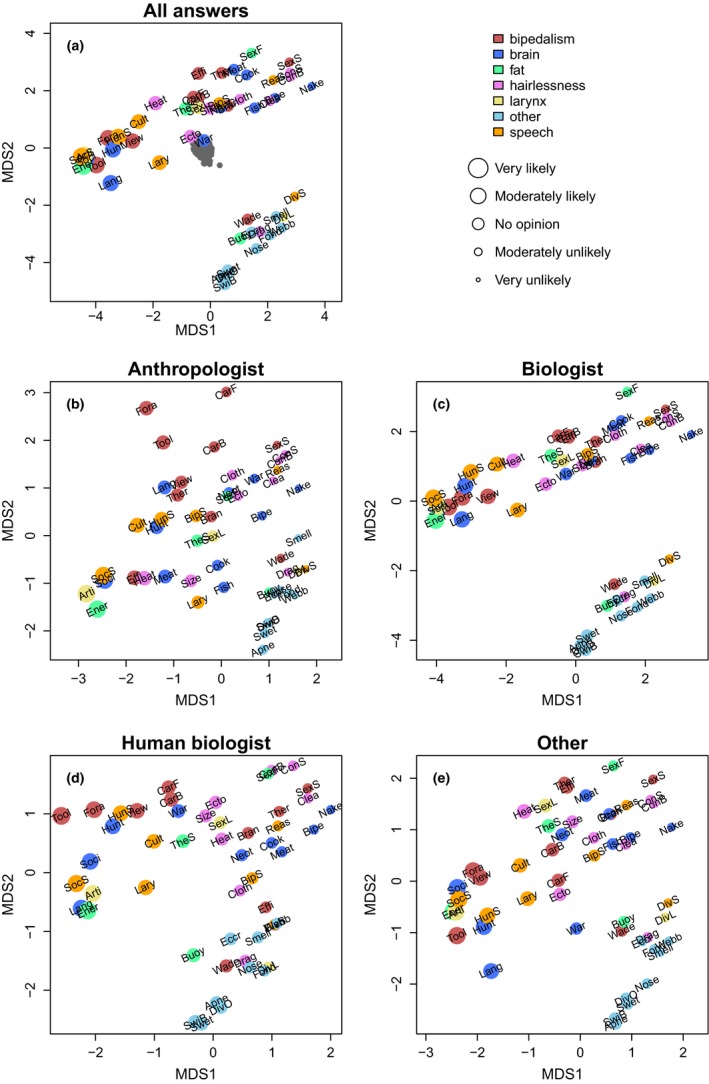
Principal coordinates analysis (PCoA) of different hypotheses proposed to explain the evolutionary origin of specific human traits. Distances between hypotheses are based on scores given by (a) all respondents, or only respondents whose main field of expertise is (b) anthropology or paleoanthropology, (c) biology, (d) human biology, or (e) other. Each colored point corresponds to one hypothesis, and the color indicates which of the traits listed in the inset the hypothesis aims to explain. Points are scaled to reflect the average credibility score given to the corresponding hypothesis by the respondents of the mentioned expertise group. The hypothesis name abbreviations are explained in Table 1. Each gray point in (a) corresponds to one respondent, whose position within the ordination space reflects the scores given to the hypotheses. For example, respondents plotted toward the bottom left part of the respondent cloud found the hypotheses plotted toward the bottom left of the hypothesis cloud more credible than the hypotheses at the top, and vice versa. More details on the respondent ordination are shown in Figure 3

Principal coordinates analysis revealed some clear patterns among the hypotheses proposed to explain the evolutionary origin of specific human traits. The most eye‐catching feature of the ordination diagram in Figure 2a is that the hypotheses got divided into two elongated groups that parallel each other but are clearly separated (the abbreviations of Fig. 2 are explained in Table 1). The smaller group contains all the hypotheses that evoke adaptation to swimming or diving as an explanatory factor for the emergence of a trait, and the larger group contains all other hypotheses, whether they refer to adaptation to a specific environment or to needs that emerge from a specific behavior. Because all the hypotheses in the smaller group refer to locomotion in water and have been included in the aquatic ape hypothesis (AAH), this group will be referred to as the water‐related or AAH group. For lack of a better unifying term, the larger group will be referred to as the dryland group.

**Table 1:** The hypotheses on the evolutionary origin of human traits that were included in an online survey to find out how popular they are among scientists. The abbreviations are used in the figures, and the full text is copied verbatim from the survey. If ambiguous, the abbreviated hypothesis is followed by a letter depicting the trait: B = bipedalism, E = encephalization (big brain), F = subcutaneous fat, N = nakedness, L = descended larynx, S = speech, O = other

Abbreviation	Bipedalism
Energy efficiency (Effi)	When covering long distances on the ground, walking or running erect on two legs is energetically more efficient than walking or running on four legs.
Thin branches (Bran)	In the canopy, walking erect facilitates using multiple supports (as in orangutans) and hence makes it possible to move on thinner branches than when brachiating or moving quadrupedally.
Wading (Wade)	In a littoral habitat, walking erect allows wading in deeper water with the nostrils above the surface (apes cross water bodies bipedally), and the same posture increases streamlining when swimming and diving for food (as in penguins).
Thermoreg B (Ther)	Walking erect helps in thermoregulation in the savanna by exposing less skin to the midday sun and more skin to cooling wind.
Better view (View)	Walking erect makes it possible to see above the savanna grass and hence spot danger from further away.
Foraging (Fora)	Walking erect makes foraging more efficient, because hands are not needed for locomotion.
Carrying food (CarF)	Walking erect makes it easier for a male to carry high‐quality food such as meat to the female and infants.
Carrying baby (CarB)	Walking erect makes it possible for a female to carry its offspring in its arms.
Tool use (Tool)	Walking erect makes it easier to use tools and weapons.
Sexual sel B (SexS)	Walking erect is favored by sexual selection, as it makes the genitals more visible.

	Big brain (encephalization)
Meat	A shift in diet toward eating more meat triggers encephalization, because meat is rich in energy.
Fish	A shift in diet toward eating more fish and other seafood triggers encephalization, because seafood is rich in both energy and the omega‐3 fatty acids that are an essential component of brain tissue.
Cooking (Cook)	The use of fire triggers encephalization, because cooking increases the nutritional value of plant foods.
Social E (Soci)	Complex social organization causes pressure for greater intelligence and hence triggers encephalization.
Hunting E (Hunt)	Collaborative hunting causes pressure for greater intelligence and hence triggers encephalization.
Language (Lang)	Spoken language causes pressure for greater intelligence and hence triggers encephalization.
Warfare (War)	Warfare causes pressure for greater intelligence and hence triggers encephalization.
Neoteny (Neot)	Encephalization is a secondary effect of neoteny (the retention of juvenile features into adulthood), which is advantageous when specialized adult morphology adapted to one environment has become maladaptive in a new environment.
Bipedalism E (Bipe)	Encephalization is triggered by bipedalism, which changes the blood circulation and provides a cooling mechanism for the larger brain.
Nakedness E (Nake)	Encephalization is triggered by nakedness, which provides a cooling mechanism for the larger brain.

	Nakedness
Skin contact baby (ConB)	Direct skin‐to‐skin contact strengthens the emotional bond between a female and its nursing offspring.
Skin contact sex (ConS)	Direct skin‐to‐skin contact makes sex more enjoyable and is favored by sexual selection.
Cleanliness (Clea)	In animals that feed messily on carrion, naked skin stays cleaner than hairy skin (or feather‐covered skin as in vultures).
Ectoparasites (Ecto)	In mammals that live in permanent nests, naked skin helps to avoid a high ectoparasite load.
Drag_thermoreg (Drag)	In mammals that live partly or entirely in water, fur is often lost because it causes drag when swimming but fails to provide efficient insulation when wet (e.g., walrus, hippopotamuses, dolphins).
Overheating (Heat)	In mammals that hunt on the savanna, naked skin dissipates heat more efficiently and reduces the risk of becoming overheated.
Body size (Size)	Large mammals can regulate their body temperature without investing in hair, and humans are relatively large compared to other primates.
Clothes (Cloth)	Once the use of clothes has become common, fur becomes unnecessary.

	Subcutaneous fat
Energy supply (Ener)	In conditions of variable food supply, subcutaneous fat can store energy for times of food scarcity, and in infants, it secures the development of the large brain.
Thermoreg buoyancy (Buoy)	In wet conditions, subcutaneous fat provides more efficient insulation than hair does, and it makes swimming easier by increasing buoyancy and streamlining of the body.
Thermoreg savanna F (TheS)	Subcutaneous fat is an adaptation to thermoregulation in the savanna, together with nakedness and sweating.
Sexual sel F (SexF)	Subcutaneous fat defines the body shape and its evolution is driven by sexual selection.

	Descended larynx
Articulation (Arti)	Articulate speech requires a descended larynx, because this makes it possible to produce a wider variety of sounds.
Sexual sel L (SexL)	A descended larynx makes the voice stronger and more impressive and can evolve through sexual selection (as in the males of some deer).
Diving L (DivL)	A descended larynx can evolve as an adaptation to diving (as in some aquatic mammals), because it makes it possible to close the air passages when under water and to inhale rapidly through the mouth when surfacing.

	Speech
Larynx S (Lary)	Speech is triggered by the descended larynx, which allows making a wider variety of sounds.
Diving S (DivS)	Speech requires voluntary breath control, which can evolve as an adaptation to diving. In water, visual and olfactory cues are inadequate and therefore liable to be replaced by vocal communication (as in whales).
Bipedalism S (BipS)	Speech requires voluntary breath control, which can evolve after bipedalism frees breathing from the constraint posed by the mechanics of locomotion.
Reassurance (Reas)	Speech provides a means for females to reassure their offspring who have to be put down while foraging.
Social S (Soci)	Social pressure for more elaborate communication triggers evolution of speech.
Hunting S (HunS)	Collective hunting requires a means of effective communication and therefore triggers evolution of speech.
Culture (Cult)	Transmitting cultural tradition (e.g., how to cope with unusually severe droughts) from one generation to the next requires a means of effective communication and therefore triggers evolution of speech.

	Other traits
Baby swimming (SwiB)	Human babies can be taken for a swim long before they can walk. They are comfortable in water and capable of holding their breath when submerged.
Nose	Unlike apes, humans have an arched nose and flexible nostrils. These help prevent water from entering the respiratory tract when diving.
Smell	Humans have a relatively weak sense of smell, as aquatic mammals often do.
Webbing (Webb)	Humans have partial webbing between their fingers and toes. Webbed feet are common among semi‐aquatic animals (such as otters and ducks), but are not found in nonhuman primates.
Eccrine glands (Eccr)	Cooling sweat is excreted from eccrine glands in humans but from apocrine glands in other primates. Apocrine glands could have lost their thermoregulatory function in human ancestors during a period when dip‐cooling replaced sweat‐cooling.
Sweating (Swet)	Humans sweat more profusely than any other primate. As this can lead to fatal loss of water and electrolytes in a few hours, the trait probably evolved in conditions of abundant water and salt supply.
Diving O (Dive)	Compared to other primates, humans are stronger swimmers and can dive both deeper and further.
Apnea (Apne)	The diving reflex (slowing down of heartbeat and oxygen usage in water) increases the resistance of the brain to apnea, and its magnitude in human divers is comparable to that in semi‐aquatic mammals such as otters and beavers.
Fond of water (Fond)	Compared to other primates, humans are unusually fond of immersing themselves in water. This is manifested in the popularity of beach holidays, swimming and bathing.

Within each of the two groups, the hypotheses got sorted by their popularity, with the average credibility score increasing toward the bottom left in Figure 2a. A tight cluster at the extreme left of the dryland group was formed by five hypotheses with high average credibility scores (4.08–4.26 on a 1–5 scale, with 1 corresponding to “very unlikely” and 5 to “very likely”). This cluster included the most popular hypothesis for the subcutaneous fat layer (energy reserve especially for the developing brain), the descended larynx (required by articulate speech), bipedalism (use of tools and weapons), speech (social pressure for elaborate communication), and the big brain (complex social organization).

This combination might be the most popular overall scenario for the origin of these traits, but the next most popular 2–3 explanations for bipedalism (freeing hands for foraging, better view over tall grass), large brain (required by either language or collaborative hunting), and speech (required by either collaborative hunting or transmitting cultural tradition; triggered by the descended larynx) also received high average credibility scores (3.53–3.96). Their proximity in ordination space indicated that they were found credible by the same respondents, which makes it difficult to identify a single most popular overall scenario. The hypotheses explaining hairlessness were not found convincing by the respondents, as even the two most popular ones (avoidance of overheating when hunting, avoidance of ectoparasites) had average credibility scores of only 3.48 and 3.17, respectively.

Eleven of the twelve most popular hypotheses were based on inherent drivers of evolution, that is, proposing that morphological traits emerged in response to selection pressure either from a novel behavior or from a pre‐existing morphological trait. Hypotheses based on selection pressure from a new kind of external environment were less popular even within the dryland group, and the credibility scores of all the hypotheses in the water‐related group were low to intermediate (2.26–2.99). The hypotheses proposing that encephalization was triggered by improved nutrition also received intermediate popularity scores, whether achieved by cooking or by increased consumption of fish or meat (all three with credibility scores in the range 2.61–2.77). The four least popular hypotheses of all (credibility scores 1.95–2.20) were based on inherent drivers operating on dry land.

**Figure 3:**
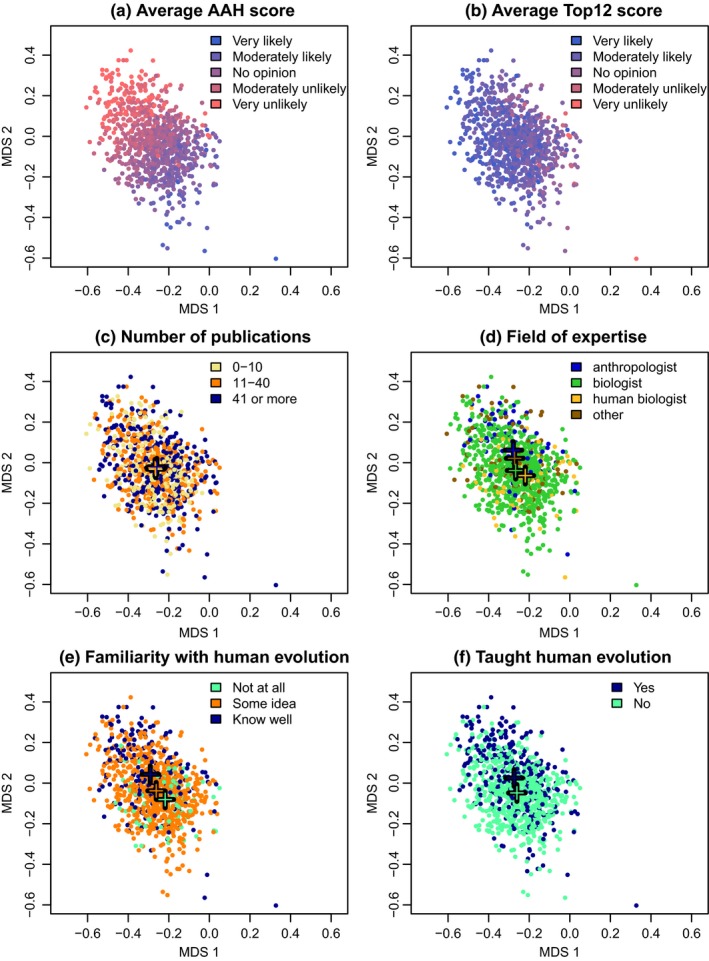
The positions of the survey respondents in the space of the principal coordinates analysis shown in Figure 2a. The ordination is the same in each panel, but colors illustrate different kinds of information related to each respondent. The colored crosses indicate the mean position of the respondents belonging to the respective subgroup. (a) Average credibility score given to the hypotheses in the water‐related group (the smaller cloud of points in Figure 2a). (b) Average score given to the 12 most popular hypotheses in Figure 2a. (c) Number of scientific publications authored or co‐authored (crosses of all three categories overlap). (d) Field of expertise. (e) Familiarity with hypotheses on human evolution. (f) Experience in teaching human evolution

The ordination results suggest that the respondents viewed the water‐related hypotheses as an ensemble whose overall credibility they assessed independently of how they scored the credibilities of the other hypotheses. This impression is strengthened when viewing the ordination of the respondents (the gray cloud in Figure 2a) in more detail (Figure 3). The main gradient among the respondents follows the average credibility score they gave for the water‐related hypotheses (Figure 2a), and this is almost perpendicular to the (less clear) gradient of average credibility scores given for the twelve most popular hypotheses (Figure 3b).

The respondents’ position in the ordination did not seem to be related with how much scientific experience they had in general, as measured with the total number of scientific publications they had authored (Figure 3c), but it was related with how much they knew about human evolution. Those having more background information on this specific topic (by self‐assessment, by main field of expertise being paleoanthropology or anthropology, or by having taught university courses on the topic) appeared to be more often plotted in the upper part of the ordination than respondents representing other backgrounds (Figure 3d–f).

The visual impressions were confirmed by statistical analyses. These were carried out separately for five different subgroupings of the hypotheses. Three of these were chosen because they formed clear groups in the ordination of Figure 2a (the dryland hypotheses, the water‐related hypotheses, the 12 most popular dryland hypotheses). The dryland hypotheses were also split into those based on environmental adaptation and those evoking behavioral drivers.

The largest effect by far on the responses was that of the field or expertise, with (paleo)anthropologists being more critical overall than representatives of any other expertise group (Table 2). The difference was especially large for the water‐related hypotheses: The average credibility score given by (paleo)anthropologists to this group of hypotheses (2.10 on the 1–5 scale) was much lower than the average score given by human biologists (3.02), with biologists (2.70), and others (2.67) being intermediate. For the dryland hypotheses, the difference between (paleo)anthropologists (2.97) and human biologists (3.22) was only 0.25 (vs. 0.92 in the case of the water‐related hypotheses), and the differences in the scores given by biologists, human biologists, and others were not statistically significant.

**Table 2:** Results of Tukey's HSD test between different subgroups of respondents (line starting with Test result ~) and their average credibility scores (standard deviation in parentheses) for different groups of hypotheses: the most popular 12 hypotheses; the dryland hypotheses (the larger hypothesis group in Figure 2a); the water‐related hypotheses (the smaller hypothesis group in Figure 2a); dryland hypotheses based on behavioural demands; dryland hypotheses based on adaptation to the external environment

Subgroup of respondents	Top 12 hypotheses, average (*SD*)	Dryland hypotheses, average (*SD*)	Water‐related hypotheses, average (*SD*)	Behavioural dryland hypotheses, average (*SD*)	Environmental dryland hypotheses, average (*SD*)
Test result ~expertise	Biol vs. anthr *** Hum vs. anthr ** Other vs. anthr **	Biol vs. anthr * Hum vs. anthr *** Other vs. anthr ***	Biol vs. anthr *** Hum vs. anthr *** Other vs. anthr *** Hum vs. biol *** Other vs. hum **	Biol vs. anthr *** hum vs. anthr *** other vs. anthr ***	Other vs. anthr ** Other vs. biol *
Anthropologist	3.71 (0.61)	2.97 (0.45)	2.11 (0.90)	3.00 (0.56)	2.95 (0.49)
Biologist	3.95 (0.56)	3.12 (0.47)	2.71 (0.82)	3.25 (0.57)	3.02 (0.50)
Human biologist	3.97 (0.70)	3.22 (0.60)	3.02 (0.81)	3.37 (0.65)	3.11 (0.64)
Other	3.98 (0.59)	3.22 (0.51)	2.67 (0.96)	3.29 (0.59)	3.16 (0.54)
Test result ~familiarity	Some vs. None *		Well vs. None *** Well vs. Some ***		
Not at all	3.81 (0.54)	3.08 (0.42)	2.92 (0.64)	3.18 (0.54)	3.01 (0.44)
I have some idea	3.96 (0.56)	3.14 (0.48)	2.76 (0.83)	3.27 (0.56)	3.05 (0.52)
I know the hypotheses well	3.87 (0.66)	3.08 (0.55)	2.27 (0.96)	3.18 (0.66)	3.00 (0.56)
Test result ~gender			Male vs. Female **		
Male	3.94 (0.59)	3.13 (0.50)	2.62 (0.88)	3.26 (0.59)	3.03 (0.54)
Female	3.91 (0.55)	3.12 (0.46)	2.81 (0.84)	3.20 (0.57)	3.05 (0.48)
Test result ~age	>60 vs. 40–49 *	>60 vs. 30–39 ** 50–59 vs. 40–49 ** >60 vs. 40–49 ***		50–59 vs. 30–39 * >60 vs. 30–39 ** 50–59 vs. 40–49 *** >60 vs. 40–49 ***	>60 vs. 30–39 * >60 vs. 40–49 *
29 or less	3.95 (0.45)	3.17 (0.36)	2.80 (0.82)	3.25 (0.46)	3.11 (0.43)
30–39	3.92 (0.51)	3.09 (0.42)	2.65 (0.84)	3.21 (0.52)	3.00 (0.46)
40–49	3.86 (0.66)	3.05 (0.53)	2.63 (0.89)	3.12 (0.61)	2.99 (0.56)
50–59	3.99 (0.63)	3.19 (0.52)	2.74 (0.89)	3.34 (0.61)	3.07 (0.57)
60 or more	4.01 (0.60)	3.24 (0.57)	2.65 (0.87)	3.39 (0.65)	3.13 (0.57)
Test result ~publications on human evolution	>41 vs. none ** >41 vs. 1–10 *		1–10 vs. none ** 11–40 vs. none *		
None	3.96 (0.58)	3.13 (0.49)	2.72 (0.84)	3.25 (0.58)	3.04 (0.52)
1–10	3.86 (0.60)	3.11 (0.50)	2.50 (0.94)	3.22 (0.58)	3.02 (0.53)
11–40	3.83 (0.53)	3.06 (0.45)	2.22 (0.95)	3.14 (0.53)	2.99 (0.52)
41 or more	3.37 (0.77)	2.86 (0.62)	2.45 (0.94)	2.96 (0.61)	2.78 (0.66)
Test result ~teaching	Yes vs. No **	Yes vs. No *	Yes vs. No **	Yes vs. No **	
Teaching: No	3.98 (0.57)	3.15 (0.49)	2.80 (0.81)	3.29 (0.57)	3.05 (0.53)
Teaching: Yes	3.84 (0.59)	3.07 (0.49)	2.39 (0.93)	3.14 (0.60)	3.01 (0.51)

The results obtained with respondent subgroups based on total number of authored peer reviewed publications and total number of authored popular science publications are not shown, because they were not associated with significantly different (*p* < .05) means in any comparisons.

***p < .001; **p < .01; *p < .05.

Overall scientific experience (as measured with the number of scientific publications authored) had no effect on the scores given to either the dryland or the water‐related hypotheses (Table 2). However, the more knowledge the respondents had on human evolution specifically (self‐assessed familiarity with the hypotheses, number of scientific publications on human evolution or experience in teaching human evolution), the lower the scores they gave to the water‐related hypotheses. Among biologists, those who knew more about human evolution were more critical than the less knowledgeable ones, and (paleo)anthropologists were more critical than human biologists with the same self‐assessed knowledge level.

When the dryland hypotheses were split into two groups depending on whether they were based on behavioral arguments or environmental adaptation, both groups obtained rather similar results. The main difference was that the behavioral hypotheses received somewhat higher average credibility scores, which reflects the fact that 10 of the 12 most popular hypotheses were based on behavior (on the other hand, so were the four least popular hypotheses).

To visualize the differences in opinion among the (paleo)anthropologists and representatives of other fields, we repeated the ordination of the hypotheses for each of the four respondent groups separately. In accordance with the fact that most respondents were biologists, the ordination based on the biologists’ data only (Figure 2c) was very similar to the ordination based on all respondents (Figure 2a). The ordination based on (paleo)anthropologists’ views (Figure 2b) differed especially in relation to the hypotheses for bipedalism: Hypotheses that explained bipedalism by foraging, tool use, or carrying were very far removed from the main cloud and toward the opposite side than the water‐related hypotheses. In addition, the average credibility scores given to the water‐related hypotheses were among the lowest of any hypotheses. This contrasted with the situation in the ordination based on human biologists’ data (Figure 2d), in which the water‐based hypotheses had intermediate credibility scores.

**Figure 4:**
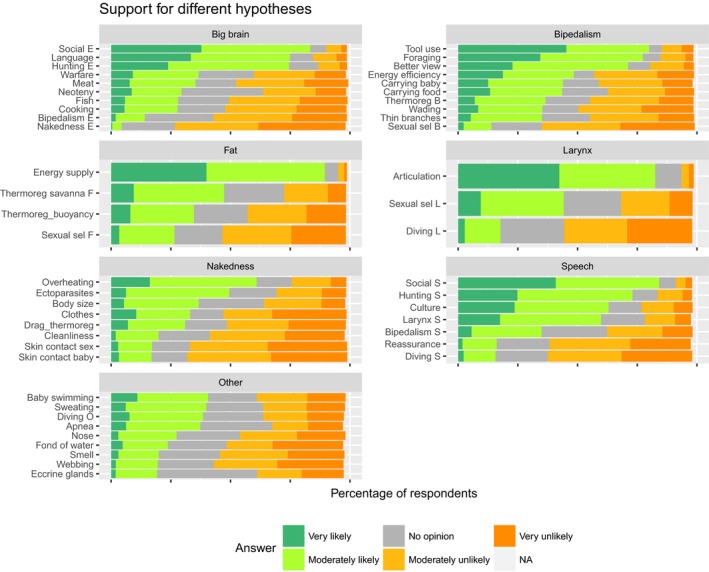
Credibility scores given by survey respondents to hypotheses that aim to explain the evolutionary origin of specific human traits. The hypotheses are sorted in order of decreasing popularity as estimated by the percentage of respondents who scored them likely (i.e., either “very likely” or “moderately likely”). Descriptions of the hypotheses as they were given in the survey are shown in Table 1

The hypotheses differed clearly from each other in the frequencies of different credibility scores, but there were some similarities in the overall pattern among those six traits for which three or more hypotheses were evaluated (Figure 4). None of the hypotheses received the “very likely” score from more than 46% of the respondents, but most traits had at least one hypothesis that was considered “very likely” by more than 23% and likely (either “very likely” or “moderately likely”) by 72%–90%. Many of the intermediately popular hypotheses divided the respondents rather evenly between those who found them likely and those who found them unlikely (the latter referring to the scores “very unlikely” and “moderately unlikely” combined).

A causal relationship between articulate speech and descended larynx was accepted by most respondents, but there was no consensus on the direction of the causality. That the larynx descended because this was required by articulate speech was found likely by 84% and very likely by 43%. At the same time, that the evolution of speech was triggered by the descended larynx was found likely by 61% and very likely by 18%. In fact, 36% of the respondents scored both directions as equally likely.

Traits in the category “other” had only one explanatory hypothesis each in the survey, and this was water‐related. All of these hypotheses received many more “very unlikely” than “very likely” scores. However, four hypotheses (that baby swimming, profuse sweating, diving ability, and magnitude of diving reflex evolved as adaptations to a semi‐aquatic way of life) received so many “moderately likely” scores that the percentage of respondents who found them likely was slightly larger than the percentage who found them unlikely (Figure 4).

**Figure 5:**
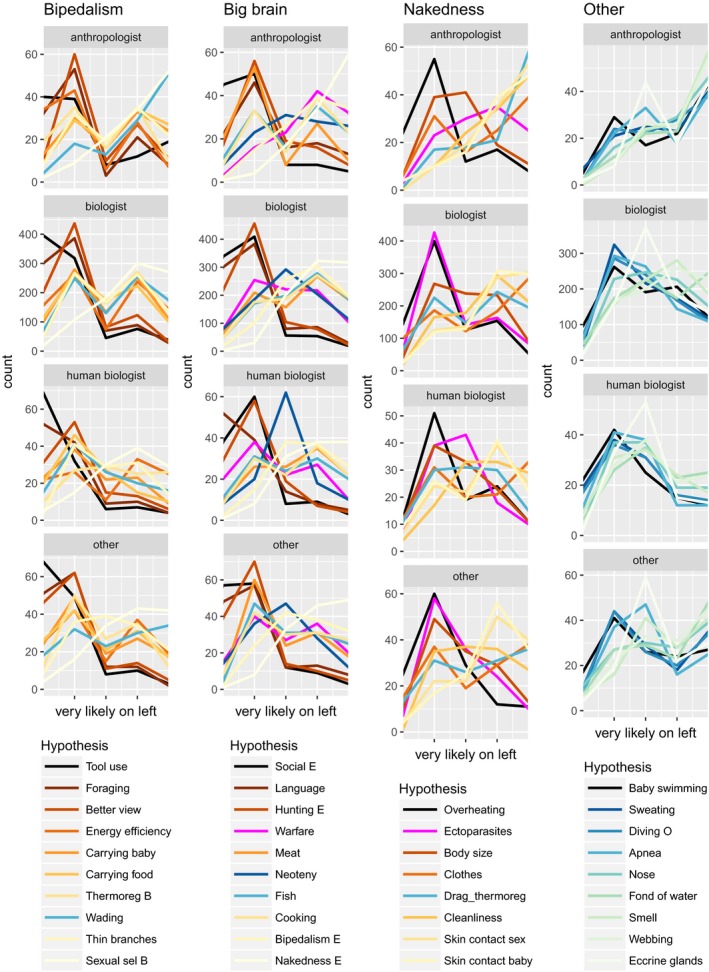
Frequencies of credibility scores given to hypotheses aiming to explain different traits (columns) by respondents of different fields of expertise (rows). In each panel, the answers are, from left to right, “very likely,” moderately likely,” “no opinion,” “moderately unlikely,” and “very unlikely.” Hypotheses that have been included in the aquatic ape hypothesis are shown in shades of blue and green. Those dryland hypotheses for which the opinions of anthropologists and other expertise groups clearly diverged are shown in magenta. The other hypotheses are in shades of brown, with darker colors given to hypotheses that received higher average credibility scores in the survey

Details on how the hypotheses were scored by respondents representing different fields of expertise are shown in Figure 5. In accordance with the statistical test results, most hypotheses received rather similar scores from respondents of all fields of expertise. However, (paleo)anthropologists were clearly more critical than representatives of the other fields in relation to several hypotheses, including: that nakedness evolved to avoid ectoparasites, that the big brain evolved because warfare caused pressure for higher intelligence, and that any traits evolved as adaptations to swimming or diving.

**Figure 6:**
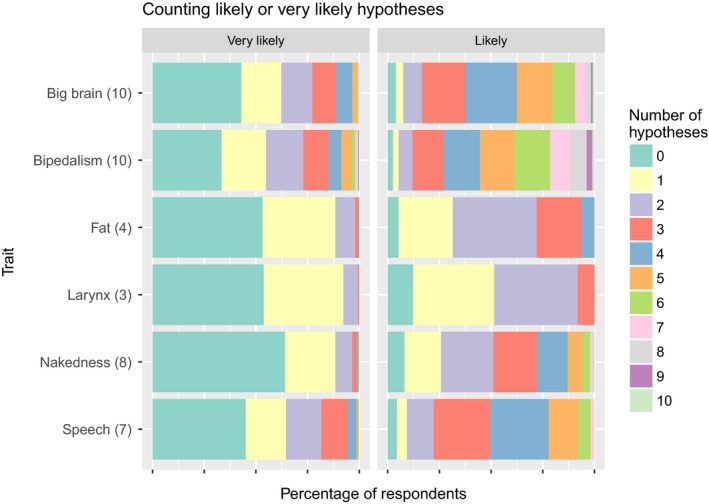
The number of hypotheses (colors) proposed to explain each human trait (rows) that each respondent found very likely (left panel) or likely (either very likely or moderately likely; right panel). The total number of hypotheses included in the survey is shown after the name of each trait

There was a lot of variation among the traits in how many of the proposed explanations the respondents found convincing (Figure 6). For any one trait, 33%–64% of the respondents did not find any of the proposed hypotheses “very likely,” while 19%–38% found exactly one and 8%–45% more than one. Ten respondents (0.8%) explained that they did not score any of the hypotheses as likely, because they do not believe that humans have evolved at all (most of them explicitly referred to special creation by God).

**Figure 7:**
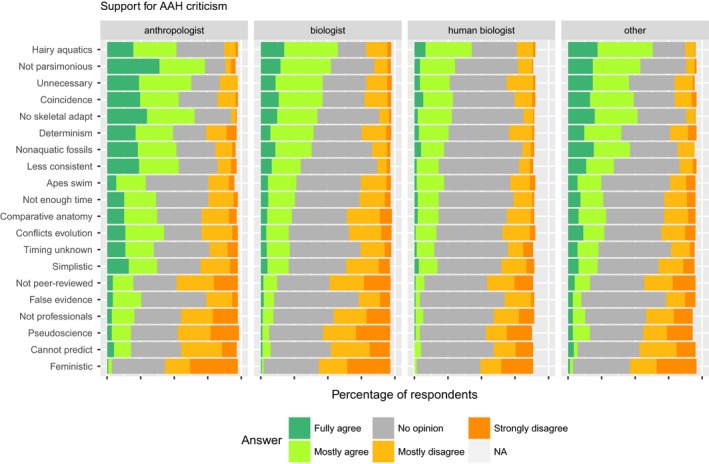
The degree to which respondents representing different expertise fields agree with critique presented against the aquatic ape hypothesis. The full description of each point of critique can be found in Table 3

The survey asked respondents’ opinions on twenty critical arguments that have been presented against the aquatic ape hypothesis. For most arguments, the modal response was “no opinion,” especially among those 43% of the respondents who had never heard of AAH before. Nevertheless, some arguments were clearly more frequently agreed with than others (Figure 7 and Table 3). The most widely accepted critique was that not all aquatic mammals have naked skin, so hairlessness cannot be considered an aquatic adaptation. In the other extreme, less than 3% of the respondents fully agreed and less than 12% mostly agreed with the critique that AAH is unscientific or not worthy of attention for the reasons given; in most cases, the number of respondents who strongly disagreed with these critiques was larger than the number who mostly or fully agreed.

**Table 3:** Points of critique presented against the aquatic ape hypothesis (AAH). The abbreviations are used in Figure 7, and the full text is copied verbatim from the survey

Abbreviation	Critique
Hairy aquatics	Not all aquatic mammals have naked skin, so hairlessness cannot be considered an aquatic adaptation.
Not parsimonious	AAH is less parsimonious than other proposed hypotheses: It has to explain both how human traits evolved in water, and how they were retained after return to land.
Unnecessary	AAH is not needed, because all human traits can be explained by terrestrial scenarios.
Coincidence	Humans may be similar to aquatic mammals in some traits, but this is only a coincidence and has no evolutionary relevance.
No skeletal adaptations	AAH is not supported by fossil evidence, because this shows no skeletal adaptations to an aquatic environment.
Determinism	A major problem with AAH is that it is based on extreme environmental determinism.
Nonaquatic fossils	AAH is contradicted by the fossil record, because this suggests a permanently nonaquatic environment.
Less consistent	AAH is internally less consistent than other proposed hypotheses.
Apes swim	According to AAH, humans should swim better than apes and have more streamlined bodies, but they do not.
Not enough time	There has not been enough time for an aquatic phase.
Comparative anatomy	AAH is merely an exercise in comparative anatomy, not a scientific hypothesis.
Conflicts evolution	AAH conflicts with what is known about evolutionary processes in general.
Timing unknown	AAH lacks credibility, because its proponents do not agree on when and where the supposed aquatic phase took place.
Simplistic	AAH is too simplistic to be taken seriously.
Not peer‐reviewed	AAH can be ignored, because it was not published in a peer‐reviewed journal, and because it is mostly discussed in forums other than scientific journals.
False evidence	AAH lacks credibility, because the evidence presented in its favor is false.
Not professionals	AAH can be ignored, because its main proponents are not professionals in the field of human evolution.
Pseudoscience	AAH is pseudoscience comparable to creationism.
Cannot predict	AAH is unscientific, because it cannot make predictions.
Feministic	AAH is unscientific, because it has been used in feministic argumentation.

## 4 DISCUSSION

The main results of our survey can be summarized as follows: (1) There was no general agreement among the respondents on why any of the uniquely human traits have evolved: None of the proposed hypotheses was universally either accepted or rejected. (2) For any individual trait, the percentage of respondents who found none of the hypotheses “very likely” was between >30% (bipedalism) and >65% (nakedness). (3) In general, opinions on the credibility of the hypotheses were independent of a person's background (gender, age, field of expertise, degree of scientific experience), but (paleo)anthropologists were clearly more critical than representatives of other fields. (4) The hypotheses that mention adaptation to swimming or diving as an explanatory factor were found much less credible by (paleo)anthropologists and slightly more credible by human biologists than by biologists and representatives of other fields. (5) Most respondents were critical about the aquatic ape hypothesis (AAH), but only a small minority considered it to be unscientific.

Of course, all conclusions based on the survey data must be considered tentative only, because the response rate was very low, and it is possible that the results are biased. Members of some subgroup might have been more likely to respond than members of some other subgroup, and the average credibility scores given to the different hypotheses by the respondents may not be representative of the opinions of all scientists in the background population. However, it is unlikely that a lack of general agreement on the drivers of trait evolution or such a clear difference in opinion between (paleo)anthropologists and others could have emerged just as a result of biased sampling.

Our results did not reveal a set of explanations that would collectively provide a coherent and popular scenario for the origin of all (or even many) human traits. Indeed, some of the hypotheses that had almost equal and rather high average credibility scores explained the same trait, whereas for other traits, no hypothesis emerged as particularly popular. Against this background, it is interesting that almost half of the respondents fully or mostly agreed with the statement that the aquatic ape hypothesis “is not needed, because all human traits can be explained by terrestrial scenarios”.

The lack of agreement on why humans evolved the traits we have today is very obvious in our results: No hypothesis was universally accepted, and for most traits, there were several almost equally popular alternative hypotheses rather than one that would generally be considered superior to the others. None of the hypotheses received the score “very likely” from more than half of the respondents or obtained an average credibility score higher than 4.26 (of 5). For hairlessness, the most popular hypothesis was thought to be “very likely” by only 16% of the respondents, and its average credibility score (3.48) was closer to 3 (which is the limit between being considered more likely than unlikely) than to 4 (moderately likely). In addition, for only two of the traits (subcutaneous fat layer and descended larynx), the most popular hypothesis was found at least moderately likely by almost all respondents at the same time as the next most popular hypothesis was found clearly less likely. This may partly reflect the fact that fewer alternative hypotheses have been proposed for these traits than for many of the others included in the survey.

Importantly, lack of agreement did not reflect just ignorance on the topic among nonspecialists, because the responses were, in general, very similar between anthropologists and respondents representing other fields of science. In fact, anthropologists were even more skeptical about all hypotheses than representatives of the other fields were. In other words, outsiders were slightly more convinced that the proposed hypotheses are plausible than those who work in the field. Maybe anthropologists (especially paleoanthropologists) are more systematically trained to be wary of just‐so‐stories (explanations of past events and processes backed up by little or no evidence) than students in nearby fields are. It is also possible that outsiders are somewhat less likely to question hypotheses proposed within an unfamiliar field. This could be because they do not feel qualified to do so, or because they have not heard of the debates that draw attention to the weaknesses of the hypotheses.

Our results conform with the widespread belief that professionals in the field of human evolution are more critical toward the aquatic ape hypothesis (AAH) than outsiders are (Langdon, 1997; Bender et al., 2012; see also nonscientific sources such as Hawks, 2005; Moore, 2012 and Wikipedia: Aquatic Ape Hypothesis: Talk). However, this did not seem to be due to overall scientific ignorance, because how respondents assessed the credibility of the hypotheses proposing adaptation to swimming or diving was independent of both their overall scientific experience level and how they assessed the credibility of the other hypotheses. Interestingly, those whose main field of expertise is human biology had the most positive attitudes toward the water‐related hypotheses, giving them an average credibility score that was as much as 0.9 units higher (on a 1–5 scale) than the average score given by (paleo)anthropologists.

The difference in average opinion between (paleo)anthropologists and other scientists can be interpreted in two opposite ways. On the one hand, those who know the field of human evolution best may be best positioned to make a justified evaluation of the validity of the alternative hypotheses. On the other hand, prior knowledge may induce one to reject unconventional hypotheses offhand merely because they challenge the established paradigms of a field (Bender et al., 2012; Klayman, 1995). Obviously, the two interpretations lead to opposite conclusions on whether or not the critical attitude of the (paleo)anthropologists can be taken as evidence that AAH is flawed. In our survey, a vast majority of the respondents who had an opinion on the issue disagreed with the statement that AAH can be ignored because its main proponents are not professionals in the field of human evolution. This was the case both overall and within each field of expertise separately, although the proportion of respondents who agreed with the statement was higher among (paleo)anthropologists than among representatives of the other fields.

In this context, it is also interesting that the respondents’ assessment of the credibility of the water‐related hypotheses did not depend on the number of scientific papers they had authored. This indicates that established scientists are no more likely to reject or accept these hypotheses than junior scientists are—unless their scientific experience relates directly to the field of human evolution. A vast majority of the respondents disagreed with the critique that AAH is unscientific. Of course, this does not mean that they would consider the explanations proposed by AAH to be correct, and indeed, all the hypotheses related to AAH received relatively low credibility scores (although not as low as the least popular dryland hypotheses).

If, for the sake of argument, we accept the most popular explanation for each trait to be the correct one, a scenario of evolution by internal drive emerges: The large brain evolved because complex social organization required higher intelligence, the subcutaneous fat layer evolved to serve as an energy reserve for the developing brain, articulate speech evolved because there was social pressure for elaborate communication, the larynx descended because this was required by articulate speech, bipedalism evolved to make the use of tools and weapons easier, and nakedness evolved to avoid overheating when hunting. For most traits, the next most popular explanation was not far behind in popularity. Most of these were also based on inherent drivers, but sometimes in the opposite temporal sequence (e.g., articulate speech was triggered by the descended larynx; large brain evolved because it was required by articulate speech). We found this result disturbing, because the overwhelming popularity of hypotheses based on inherent drivers gives the impression that human evolution is generally thought to have been goal‐directed. This would be in conflict with the current understanding (explained in every evolutionary biology textbook) that evolution has no foresight.

Overall, the survey revealed no general agreement among the respondents: None of the proposed hypotheses on why specific uniquely human traits have evolved was universally either accepted or rejected. Nevertheless, identifying and quantifying what is *not* generally known and agreed upon can be useful in itself, as it may help to focus future research on answering the most important open questions. Clearly, there is still a long way to go before the question “why are humans so different from other primates” has been answered in a comprehensive and generally satisfactory way.

## DATA ACCESSIBILITY

Data are available from the Dryad Digital Repository: https://doi.org/10.5061/dryad.s9r98

## CONFLICT OF INTEREST

None declared.

## AUTHOR CONTRIBUTIONS

HT designed and conducted the survey and led the writing. All authors discussed the results and planned the data analyses together. The R code used to analyze the data and draw the figures was written by MT with contributions from JT.
