# Supplementary material for: How scientists perceive the evolutionary origin of human traits: Results of a survey study
Source: Ecol Evol. 2018 Feb 26;8(6):3518–33. doi: 10.1002/ece3.3887 (PMC5869357; doi:10.1002/ece3.3887)
Supplement: Supplementary file 1 [file ECE3-8-3518-s001.pdf]

Appendix 1. List of the journals used to find invitees for the survey.

In all subject categories, the same cutoff limit for inclusion of a journal was applied:
ISI Impact Factor ≥ 1.0

Journals of the following ISI categories were included, if they fulfilled the additional criterion specified in parentheses:

Palaeontology (all)
Zoology (all except journals whose focus excluded mammals)
Biology (excluding narrow specialities and biomedical journals)
Ecology (all journals that covered animal ecology, if not restricted to conservation or biogeography)
Evolutionary biology (excluding those restricted to non-mammalian taxa or molecular/genomic studies)

Anthropology (if they showed evidence of covering human evolution, not just culture)
Psychology, biological (if they showed evidence of covering evolutionary aspects)
Anatomy & morphology (if they showed evidence of covering evolutionary aspects and/or comparisons among species)
Behavioral sciences (if focus included wild animals and/or evolutionary aspects)

Dermatology (selected a few broad-scope top journals)
Neurosciences (some broad-scope journals)
Nutrition & Dietetics (some broad-scope journals)
Physiology (some broad-scope journals)
Respiratory system (some broad-scope journals)
Sport sciences (some broad-scope journals)

Searched in:
Science Citation Index Expanded (SCI-EXPANDED) --1900-present
Social Sciences Citation Index (SSCI) --1900-present
Date range: 1.1.2010-31.12.2012
Article type: Article or Review
Found 72861 articles

ISSN Abbreviated journal title
0873-9749 ACTA ETHOL
1146-609X ACTA OECOL
0567-7920 ACTA PALAEONTOL POL
0001-7272 ACTA ZOOL-STOCKHOLM
0065-2504 ADV ECOL RES
0065-3454 ADV STUD BEHAV
1042-0533 AM J HUM BIOL
0002-9483 AM J PHYS ANTHROPOL
0363-6135 AM J PHYSIOL-HEART C
1040-0605 AM J PHYSIOL-LUNG C
1931-857X AM J PHYSIOL-RENAL
0275-2565 AM J PRIMATOL
0003-0147 AM NAT
1932-8486 ANAT REC
0003-3472 ANIM BEHAV
1435-9448 ANIM COGN
0940-9602 ANN ANAT
0301-4460 ANN HUM BIOL
0753-3969 ANN PALEONTOL
0003-455X ANN ZOOL FENN
0084-6570 ANNU REV ANTHROPOL
1543-592X ANNU REV ECOL EVOL S

1941-1405 ANNU REV MAR SCI
0147-006X ANNU REV NEUROSCI
0199-9885 ANNU REV NUTR
0066-4278 ANNU REV PHYSIOL
0918-7960 ANTHROPOL SCI
1864-7790 AQUAT BIOL
1386-2588 AQUAT ECOL
1442-9985 AUSTRAL ECOL
0003-0090 B AM MUS NAT HIST
1214-1119 B GEOSCI
1439-1791 BASIC APPL ECOL
1744-9081 BEHAV BRAIN FUNCT
0166-4328 BEHAV BRAIN RES
0140-525X BEHAV BRAIN SCI
1045-2249 BEHAV ECOL
0340-5443 BEHAV ECOL SOCIOBIOL
0376-6357 BEHAV PROCESS
0005-7959 BEHAVIOUR
1726-4170 BIOGEOSCIENCES
0006-3185 BIOL BULL-US
0024-4066 BIOL J LINN SOC
1744-9561 BIOL LETTERS
1464-7931 BIOL REV
0006-3568 BIOSCIENCE
0006-3606 BIOTROPICA
1741-7007 BMC BIOL
1471-2148 BMC EVOL BIOL
0006-8950 BRAIN
0006-8977 BRAIN BEHAV EVOLUT
0093-934X BRAIN LANG
0165-0173 BRAIN RES REV
1863-2653 BRAIN STRUCT FUNCT
0007-1145 BRIT J NUTR
0008-4301 CAN J ZOOL
0009-7322 CIRCULATION
0748-3007 CLADISTICS
1095-6433 COMP BIOCHEM PHYS A
1383-4517 CONTRIB ZOOL
0010-9452 CORTEX
1631-0683 CR PALEVOL
0195-6671 CRETACEOUS RES
0011-3204 CURR ANTHROPOL
0949-944X DEV GENES EVOL
0378-3782 EARLY HUM DEV
0906-7590 ECOGRAPHY
1476-945X ECOL COMPLEX
1461-023X ECOL LETT
0012-9615 ECOL MONOGR
0912-3814 ECOL RES
0012-9658 ECOLOGY
0394-9370 ETHOL ECOL EVOL
0179-1613 ETHOLOGY
1436-6207 EUR J NUTR
0903-1936 EUR RESPIR J
0940-6719 EUR SPINE J
1060-1538 EVOL ANTHROPOL
1752-4571 EVOL APPL
0071-3260 EVOL BIOL
1520-541X EVOL DEV
0269-7653 EVOL ECOL
1522-0613 EVOL ECOL RES
1090-5138 EVOL HUM BEHAV
0014-3820 EVOLUTION
0091-6331 EXERC SPORT SCI REV
0172-9179 FACIES
0015-5713 FOLIA PRIMATOL
1435-1943 FOSS REC
1662-5129 FRONT NEUROANAT
1742-9994 FRONT ZOOL
0269-8463 FUNCT ECOL
0966-6362 GAIT POSTURE
1555-8932 GENES NUTR
1472-4677 GEOBIOLOGY
0016-6995 GEOBIOS-LYON
1280-9659 GEODIVERSITAS
1695-6133 GEOL ACTA
0147-9563 HEART LUNG
0018-067X HEREDITY
0891-2963 HIST BIOL
0018-7143 HUM BIOL
0300-7839 HUM ECOL
0167-9457 HUM MOVEMENT SCI
1045-6767 HUM NATURE-INT BIOS
1479-5868 INT J BEHAV NUTR PHY
0307-0565 INT J OBESITY
1747-7166 INT J PEDIATR OBES
0164-0291 INT J PRIMATOL
1526-484X INT J SPORT NUTR EXE
1540-7063 INTEGR COMP BIOL
1749-4877 INTEGR ZOOL
0190-9622 J AM ACAD DERMATOL
0021-8782 J ANAT
0021-8790 J ANIM ECOL
1827-4765 J ANTHROPOL SCI
8750-7587 J APPL PHYSIOL
0305-4403 J ARCHAEOL SCI
0250-5991 J BIOSCIENCES
0021-9967 J COMP NEUROL

0340-7594 J COMP PHYSIOL A
0174-1578 J COMP PHYSIOL B
0735-7036 J COMP PSYCHOL
0923-1811 J DERMATOL SCI
0289-0771 J ETHOL
1010-061X J EVOLUTION BIOL
0022-0949 J EXP BIOL
0022-0981 J EXP MAR BIOL ECOL
0097-7403 J EXP PSYCHOL ANIM B
1932-5223 J EXP ZOOL PART A
1552-5007 J EXP ZOOL PART B
0096-1191 J FORAMIN RES
0047-2484 J HUM EVOL
0952-3871 J HUM NUTR DIET
1550-2783 J INT SOC SPORT NUTR
0022-202X J INVEST DERMATOL
1064-7554 J MAMM EVOL
0022-2372 J MAMMAL
0047-2565 J MED PRIMATOL
0362-2525 J MORPHOL
0022-3166 J NUTR
0022-3360 J PALEONTOL
0267-8179 J QUATERNARY SCI
1477-2019 J SYST PALAEONTOL
0306-4565 J THERM BIOL
0266-4674 J TROP ECOL
0272-4634 J VERTEBR PALEONTOL
0952-8369 J ZOOL
0947-5745 J ZOOL SYST EVOL RES
0024-1164 LETHAIA
1616-5047 MAMM BIOL
0305-1838 MAMMAL REV
0025-3162 MAR BIOL
1745-1000 MAR BIOL RES
0173-9565 MAR ECOL-EVOL PERSP
0171-8630 MAR ECOL-PROG SER
1323-1650 MAR FRESHWATER RES
0824-0469 MAR MAMMAL SCI
0377-8398 MAR MICROPALEONTOL
1740-8695 MATERN CHILD NUTR
0195-9131 MED SCI SPORT EXER
1471-003X NAT REV NEUROSCI
0896-6273 NEURON
1743-7075 NUTR METAB
0271-5317 NUTR RES
0954-4224 NUTR RES REV
0029-6643 NUTR REV
0899-9007 NUTRITION
1930-7381 OBESITY
0029-8549 OECOLOGIA
0030-1299 OIKOS
1439-6092 ORG DIVERS EVOL
0962-8452 P ROY SOC B-BIOL SCI
0031-0182 PALAEOGEOGR PALAEOCL
0375-0442 PALAEONTOGR ABT A
0375-0299 PALAEONTOGR ABT B
1935-3952 PALAEONTOL ELECTRON
0031-0220 PALAEONTOL Z
0031-0239 PALAEONTOLOGY
0883-1351 PALAIOS
0094-8373 PALEOBIOLOGY
0883-8305 PALEOCEANOGRAPHY
0962-8436 PHILOS T R SOC B
1522-2152 PHYSIOL BIOCHEM ZOOL
0031-9333 PHYSIOL REV
1548-9213 PHYSIOLOGY
1544-9173 PLOS BIOL
0032-8332 PRIMATES
0079-6123 PROG BRAIN RES
0163-7827 PROG LIPID RES
0301-0082 PROG NEUROBIOL
0033-5770 Q REV BIOL
0033-5894 QUATERNARY RES
0277-3791 QUATERNARY SCI REV
0217-2445 RAFFLES B ZOOL
1465-993X RESP RES
1323-7799 RESPIROLOGY
0034-6667 REV PALAEOBOT PALYNO
0038-6804 SPEC PAP PALAEONTOL
0362-2436 SPINE
1063-5157 SYST BIOL
1547-139X STRATIGRAPHY
1364-6613 TRENDS COGN SCI
0169-5347 TRENDS ECOL EVOL
0166-2236 TRENDS NEUROSCI
0939-6314 VEG HIST ARCHAEOBOT
0096-848X YEARB PHYS ANTHROPOL
0044-5231 ZOOL ANZ
0024-4082 ZOOL J LINN SOC-LOND
0300-3256 ZOOL SCR
0944-2006 ZOOLOGY
0720-213X ZOOMORPHOLOGY
