# Supplementary material for: How scientists perceive the evolutionary origin of human traits: Results of a survey study
Source: Ecol Evol. 2018 Feb 26;8(6):3518–33. doi: 10.1002/ece3.3887 (PMC5869357; doi:10.1002/ece3.3887)
Supplement: Supplementary file 2 [file ECE3-8-3518-s002.pdf]

Appendix 2. PDF version of the questions presented to respondents in the online survey.

Evolutionary origin of human traits

Please start by giving some background information about yourself

1. Gender

- ○ Male ○ Female

2. Age

- ○ 29 or less
- ○ 30-39
- ○ 40-49
- ○ 50-59
- ○ 60 or more

3. Country of residence

4. Academic education

Indicate highest degree earned

- ○ None
- ○ Bachelor's degree
- ○ Master's degree
- ○ Doctor's degree

5. Main fields of expertise

- ☐ Anthropology or archaeology
- ☐ Biology (animal physiology, anatomy or morphology)
- ☐ Biology (ecology)
- ☐ Biology (evolution)

☐ Biology (genetics or molecular biology)

☐ Biology (other, please specify) ______

☐ Geology

☐ Human cardiovascular or respiratory system

☐ Human musculoskeletal system

☐ Human nervous system

☐ Human nutrition

☐ Other aspects of human biology (please specify) ______

☐ Paleoanthropology

☐ Paleontology

☐ Other, please specify ______

6. Scientific experience: How many publications have you (co)authored?

These can be on any scientific topic.

	none	1-10	11-40	41 or more
Peer reviewed articles in scientific journals	○	○	○	○
Articles or books targeted at the general public	○	○	○	○

7. Scientific experience: How many publications have you (co)authored on human evolution?

	none	1-10	11-40	41 or more
Peer reviewed articles in scientific journals	○	○	○	○
Articles or books targeted at the general public	○	○	○	○

8. Have you taught university courses that cover the evolutionary origin of humans?

○ Yes ○ No

9. How familiar are you with the existing evolutionary hypotheses on what caused humans to differentiate from apes?

○ Not at all

○ I have some idea

○ I know the hypotheses well

The following six questions focus on human traits whose evolutionary origin has been

extensively discussed. For each trait, a list is provided with brief summaries of hypotheses that have been proposed in the scientific literature to explain it. Please indicate how likely you find each explanation. In case the hypothesis you find the most likely is missing from a list, you can add a comment on this at the end of the survey.

10. Humans are the only mammals whose main mode of locomotion is to walk on their hind legs with the spine held erect. This has required extensive structural and functional changes to the body, and an improved balance-keeping mechanism. How likely do you think it is that the following might cause obligate bipedalism to evolve in a primate?

	Very likely	Moderately likely	No opinion	Moderately unlikely	Very unlikely
When covering long distances on the ground, walking or running erect on two legs is energetically more efficient than walking or running on four legs.	○	○	○	○	○
In the canopy, walking erect facilitates facilitates using multiple supports (as in orangutans) and hence makes it possible to move on thinner branches than when brachiating or moving quadrupedally.	○	○	○	○	○
In a littoral habitat, walking erect allows wading in deeper water with the nostrils above the surface (apes cross water bodies bipedally), and the same posture increases streamlining when swimming and diving for food (as in penguins).	○	○	○	○	○
Walking erect helps in thermoregulation in the savanna by exposing less skin to the midday sun and more skin to cooling wind.	○	○	○	○	○
Walking erect makes it possible to see see above the savanna grass and hence spot danger from further away.	○	○	○	○	○
Walking erect makes foraging more efficient, because hands are not needed for locomotion.	○	○	○	○	○
Walking erect makes it easier for a male to carry high-quality food such as meat to the female and infants.	○	○	○	○	○
Walking erect makes it possible for a female to carry its offspring in its arms.	○	○	○	○	○

Walking erect makes it easier to use tools and weapons.	○	○	○	○	○
Walking erect is favored by sexual selection, as it makes the genitals more visible.	○	○	○	○	○

11. Human brains are larger than those of any other primate. Brains are energetically costly to build and maintain, and a big head makes giving birth dangerous both to the mother and the baby. How likely do you think it is that the following might cause encephalization (evolution of a big brain) in a primate?

	Very likely	Moderately likely	No opinion	Moderately unlikely	Very unlikely
A shift in diet towards eating more meat triggers encephalization, because meat is rich in energy.	○	○	○	○	○
A shift in diet towards eating more fish fish and other seafood triggers encephalization, because seafood is rich in both energy and the omega-3 fatty acids that are an essential component of brain tissue.	○	○	○	○	○
The use of fire triggers encephalization, because cooking increases the nutritional value of plant foods.	○	○	○	○	○
Complex social organization causes pressure for greater intelligence and hence triggers encephalization.	○	○	○	○	○
Collaborative hunting causes pressure for greater intelligence and hence triggers encephalization.	○	○	○	○	○
Spoken language causes pressure for greater intelligence and hence triggers encephalization.	○	○	○	○	○
Warfare causes pressure for greater intelligence and hence triggers encephalization.	○	○	○	○	○
Encephalization is a secondary effect of neoteny (the retention of juvenile features into adulthood), which is advantageous when specialized adult morphology adapted to one environment has become maladaptive in a new environment.	○	○	○	○	○
Encephalization is triggered by bipedalism, which changes the blood circulation and provides a cooling mechanism for the larger brain.	○	○	○	○	○

Encephalization is triggered by nakedness, which provides a cooling mechanism for the larger brain.	○	○	○	○	○

12. Almost all mammals have fur, which provides protection against physical damage, sunburn and extreme temperatures. Humans are unique among primates in having a functionally naked skin. How likely do you think it is that the following might cause body hair to be lost in a primate?

	Very likely	Moderately likely	No opinion	Moderately unlikely	Very unlikely
Direct skin-to-skin contact strengthens strengthens the emotional bond between a female and its nursing offspring.	○	○	○	○	○
Direct skin-to-skin contact makes sex more enjoyable, and is favored by sexual selection.	○	○	○	○	○
In animals that feed messily on carrion, naked skin stays cleaner than hairy skin (or feather-covered skin as in vultures).	○	○	○	○	○
In mammals that live in permanent nests, naked skin helps to avoid a high ectoparasite load.	○	○	○	○	○
In mammals that live partly or entirely entirely in water, fur is often lost because it causes drag when swimming but fails to provide efficient insulation when wet (e.g. walrus, hippopotamuses, dolphins).	○	○	○	○	○
In mammals that hunt on the savanna, naked skin dissipates heat more efficiently and reduces the risk of becoming overheated.	○	○	○	○	○
Large mammals can regulate their body temperature without investing in hair, and humans are relatively large compared to other primates.	○	○	○	○	○
Once the use of clothes has become common, fur becomes unnecessary.	○	○	○	○	○

13. Human skin is unique among primates in being attached to a subcutaneous fat layer that can become so thick as to impede physical movement. Human babies are energetically costly to the mother, because they are much more plump than those of other primates at birth and accumulate even more fat during lactation. How likely do you think it is that the following might cause a subcutaneous fat layer to evolve in a primate?

	Very likely	Moderately likely	No opinion	Moderately unlikely	Very unlikely
In conditions of variable food supply, subcutaneous fat can store energy for times of food scarcity, and in infants it secures the development of the large brain.	○	○	○	○	○
In wet conditions, subcutaneous fat provides more efficient insulation than hair does, and it makes swimming easier by increasing buoyancy and streamlining of the body.	○	○	○	○	○
Subcutaneous fat is an adaptation to thermoregulation in the savanna, together with nakedness and sweating.	○	○	○	○	○
Subcutaneous fat defines the body shape and its evolution is driven by sexual selection.	○	○	○	○	○

14. Humans are unique among primates in having a descended larynx (the head of the windpipe is permanently situated in the throat rather than in the nasal cavity). This causes a risk of choking (potentially fatally) on food or one's own tongue. How likely do you think it is that the following might cause a descended larynx to evolve in a primate?

	Very likely	Moderately likely	No opinion	Moderately unlikely	Very unlikely
Articulate speech requires a descended larynx, because this makes it possible to produce a wider variety of sounds.	○	○	○	○	○
A descended larynx makes the voice stronger and more impressive, and can evolve through sexual selection (as in the males of some deer).	○	○	○	○	○
A descended larynx can evolve as an adaptation to diving (as in some aquatic mammals), because it makes it possible to close the air passages when under water and to inhale rapidly through the mouth when surfacing.	○	○	○	○	○

15. Some apes have learned to communicate through signs and symbols, but none has learned to speak. How likely do you think it is that the following might cause a primate to shift from visual and olfactory communication to verbal speech?

Very	Moderately	No	Moderately	Very

	likely	likely	opinion	unlikely	unlikely
Speech is triggered by the descended larynx, which allows making a wider variety of sounds.	○	○	○	○	○
Speech requires voluntary breath control, which can evolve as an adaptation to diving. In water, visual and olfactory cues are inadequate and therefore liable to be replaced by vocal communication (as in whales).	○	○	○	○	○
Speech requires voluntary breath control, which can evolve after bipedalism frees breathing from the constraint posed by the mechanics of locomotion.	○	○	○	○	○
Speech provides a means for females to reassure their offspring who have to be put down while foraging.	○	○	○	○	○
Social pressure for more elaborate communication triggers evolution of speech.	○	○	○	○	○
Collective hunting requires a means of effective communication and therefore triggers evolution of speech.	○	○	○	○	○
Transmitting cultural tradition (e.g., how to cope with unusually severe droughts) from one generation to the next requires a means of effective communication and therefore triggers evolution of speech.	○	○	○	○	○

16. The origins of the following human traits have been rarely discussed in the scientific literature, but the proposal has been made that they evolved because the ancestors of humans were adapting to a semi-aquatic way of life at some stage after the separation of the human and ape lineages. Please indicate how likely you find this explanation for each trait.

	Very likely	Moderately likely	No opinion	Moderately unlikely	Very unlikely
Human babies can be taken for a swim swim long before they can walk. They are comfortable in water and capable of holding their breath when submerged.	○	○	○	○	○
Unlike apes, humans have an arched nose and flexible nostrils. These help prevent water from entering the respiratory tract when diving.	○	○	○	○	○
Humans have a relatively weak sense					

of smell, as aquatic mammals often do.	○	○	○	○	○
Humans have partial webbing between their fingers and toes. Webbed feet are common among semi-aquatic animals (such as otters and ducks), but are not found in non-human primates.	○	○	○	○	○
Cooling sweat is excreted from eccrine glands in humans but from apocrine glands in other primates. Apocrine glands could have lost their thermoregulatory function in human ancestors during a period when dip-cooling replaced sweat-cooling.	○	○	○	○	○
Humans sweat more profusely than any other primate. Since this can lead to fatal loss of water and electrolytes in a few hours, the trait probably evolved in conditions of abundant water and salt supply.	○	○	○	○	○
Compared to other primates, humans are stronger swimmers and can dive both deeper and further.	○	○	○	○	○
The diving reflex (slowing down of heartbeat and oxygen usage in water) increases the resistance of the brain to apnea, and its magnitude in human divers is comparable to that in semi-aquatic mammals such as otters and beavers.	○	○	○	○	○
Compared to other primates, humans are unusually fond of immersing themselves in water. This is manifested in the popularity of beach holidays, swimming and bathing.	○	○	○	○	○

17. If you know of hypotheses that explain the evolutionary origin of these traits on dry land, please briefly outline each hypothesis below.

Baby swimming

Arched nose and flexible nostrils

Weak sense of smell

Webbing between fingers and toes

Sweating through eccrine rather than apocrine glands

Profuse sweating

Swimming and diving ability

Diving reflex

Fondness for swimming and bathing

18. The proposal that all the human traits mentioned in the previous questions could have evolved as adaptations to a coastal habitat and a semi-aquatic way of life has been dubbed the Aquatic Ape Hypothesis (AAH). To what degree do you agree with the following criticism that has been targeted against this idea?

	Fully agree	Mostly agree	No opinion	Mostly disagree	Strongly disagree
AAH conflicts with what is known about evolutionary processes in general.					
A major problem with AAH is that it is based on extreme environmental determinism.					
AAH is not needed, because all human traits can be explained by terrestrial scenarios.					
AAH is merely an exercise in comparative comparative anatomy, not a scientific hypothesis.					
Not all aquatic mammals have naked skin, so hairlessness cannot be considered an aquatic adaptation.					
Humans may be similar to aquatic mammals in some traits, but this is only a coincidence and has no evolutionary relevance.					
According to AAH, humans should swim better than apes and have more streamlined bodies, but they do not.					
AAH lacks credibility, because the evidence presented in its favor is false.					

AAH is not supported by fossil evidence, because this shows no skeletal adaptations to an aquatic environment.

AAH is contradicted by the fossil record, because this suggests a permanently non-aquatic environment.

AAH lacks credibility, because its proponents do not agree on when and where the supposed aquatic phase took place.

There has not been enough time for an aquatic phase.

AAH is too simplistic to be taken seriously.

AAH is less parsimonious than other proposed hypotheses: it has to explain both how human traits evolved in water, and how they were retained after return to land.

AAH is internally less consistent than other proposed hypotheses.

AAH is unscientific, because it cannot make predictions.

AAH is unscientific, because it has been used in feministic argumentation.

AAH can be ignored, because it was not published in a peer reviewed journal, and because it is mostly discussed in forums other than scientific journals.

AAH can be ignored, because its main proponents are not professionals in the field of human evolution.

AAH is pseudoscience comparable to creationism.

19. If you find that important arguments that disprove the Aquatic Ape Hypothesis are missing from the above list, please outline them here:

20. If you indicated that the Aquatic Ape Hypothesis is less parsimonious or internally less consistent than another proposed hypothesis, please outline that superior hypothesis here:

21. Had you heard of the Aquatic Ape Hypothesis before this survey? *

The idea is also known as 'the Waterside Hypotheses of Human Evolution' and the 'Aquatic Ape Theory'.

- ( ) No
- (•) Yes

22. How much have the following sources contributed to what you know about the Aquatic Ape Hypothesis?

	Considerably	A little	Not at all
Articles in scientific journals	( )	( )	( )
Books by Elaine Morgan	( )	( )	( )
Books by other authors	( )	( )	( )
Articles or programs in popular media (press, TV, radio) radio)	( )	( )	( )
University courses on human evolution	( )	( )	( )
Personal communication from someone who knew the hypothesis	( )	( )	( )
Blogs or other personal web pages	( )	( )	( )
Wikipedia	( )	( )	( )

23. How common were the following attitudes towards the Aquatic Ape Hypothesis (AAH) in those sources?

	Common	Rare	Not seen
AAH was rejected as implausible.	( )	( )	( )
AAH was mentioned or described, but no opinion on its validity was expressed.	( )	( )	( )
AAH was found more plausible than alternative hypotheses.	( )	( )	( )

24. If you wish, you can comment on one or more hypotheses concerning the origin of human traits here:

Save answers to continue later

0% completed
